# Dietary cholesterol, female gender and n-3 fatty acid deficiency are more important factors in the development of non-alcoholic fatty liver disease than the saturation index of the fat

**DOI:** 10.1186/1743-7075-8-4

**Published:** 2011-01-24

**Authors:** Tine M Comhair, Sonia C Garcia Caraballo, Cornelis HC Dejong, Wouter H Lamers, S Eleonore Köhler

**Affiliations:** 1 Department of Anatomy & Embryology, Maastricht University, Maastricht, The Netherlands; 2 Department of General Surgery, Maastricht University, Maastricht, The Netherlands; 3 NUTRIM School for Nutrition, Toxicology and Metabolism, Maastricht University, Maastricht, The Netherlands; 4 Tytgat Institute for Liver and Intestinal Research, Academic Medical Center, University of Amsterdam, Amsterdam, The Netherlands; 5 Nutrigenomics Consortium, Top Institute Food and Nutrition, Wageningen, The Netherlands

## Abstract

**Background:**

The central feature of NAFLD is a disturbed fatty-acid metabolism with hepatic lipid accumulation. However, the factors that determine the severity of NAFLD, including the role of nutrition, gender, and plasma lipid levels, remain to be determined.

**Methods:**

High-fat diets (42 en% fat), containing 0.2% cholesterol, were fed to male and female wild-type and hyperlipidemic *APOE2ki *C57BL/6J mice for three weeks. The fats were, in order of decreasing saturation, fractionated palm fat (fPF; ~95%), cocoa butter (CB; ~60%), olive oil (OO; ~15%), sunflower oil (SO; ~12%), and high-oleic-acid sunflower oil (hoSO; ~7%). Plasma and liver triglycerides (concentration and composition), liver inflammation (*Ccl2*, *Cd68*, *Tnf-α *mRNA), and infiltration of macrophages (Cd68, Cd11b immunohistochemistry) and neutrophils (Mpo) were quantified.

**Results:**

Addition of cholesterol to a low-fat diet decreased plasma HDL and increased (V)LDL levels in APOE2ki mice. Plasma cholesterol levels in female, but not male APOE2ki mice correlated significantly with inflammation. Kupffer cells of inflamed livers were swollen. Wild-type mice refused the highly saturated fPF diet. The high-fat CB, OO, and SO diets induced hyperglycemia and a 2-fold increase in hepatic fat content in male, but not female wild-type mice (in females, hepatic fat content was similar to that in males fed a high-fat diet). All high-fat diets induced macrovesicular setatosis. APOE2ki mice were protected against high-fat diet-induced steatosis and hyperglycemia, except when fed a hoSO diet. This diet caused a 5-fold increase in liver triglyceride and mead-acid content, and an increased expression of lipogenic genes, suggesting a deficiency in poly-unsaturated fatty acids. Irrespective of the composition of the high-fat diet, oleic acid was the main triglyceride component of liver fat in wild-type and APOE2ki mouse livers. Liver inflammation was dependent on genotype (APOE2ki > wild type), gender (female > male), and cholesterol content (high > low) of the diet, but not on dietary fat composition.

**Conclusions:**

Dietary cholesterol plays a determining, independent role in inflammation, especially in female mice. The fatty-acid saturation of the diet hardly affected hepatic steatosis or inflammation.

## Background

Non-alcoholic fatty liver disease (NAFLD) refers to the non-physiological accumulation of fat in the liver. The increased prevalence of NAFLD is a concomitant effect of the increased incidence of obesity and a feature of the metabolic syndrome [1]. The central feature of NAFLD is a disturbed fatty-acid metabolism with hepatic lipid accumulation. Lipid accumulation alone is benign, but may sensitize the liver to hepatic injury (2-hit hypothesis) [2]. Alternatively, harmful side products (e.g. (lyso-) phosphatidic acid, lysophosphatidylcholine, diacylglycerols, and ceramides) of enhanced fatty-acid metabolism may be responsible for liver damage (lipotoxicity hypothesis) [3]. However, the cause(s) and circumstances that determine the severity of NAFLD, including the role of nutrition, gender, and plasma lipid levels, remain to be determined.

The limited progress in clarifying the causes of NASH may at least partly be due to the diversity in the reported effects of different dietary fats in various models. Saturated fats, especially palmitic acid, may facilitate the progression of NAFLD to NASH by inducing endoplasmic reticulum (ER) stress and/or the synthesis of ceramides [4]. Monounsaturated fats (MUFA), such as olive oil, were, on the other hand, reported to be more effective in decreasing steatosis in the methionine-choline deficient model than fish oil or butter fat [5]. Fish oil, in turn, appears to have more reproducible effects than polyunsaturated plant oils [6,7]. In fact, plant PUFAs appear not to be more beneficial in rodents with respect to steatosis than saturated fats [4,5,8]. Most of these studies were carried out in (Wistar) rats [6,9], but such data are not yet available for a mouse strain that is prone to become obese on a high-fat diet, such as WT mice, nor in a model for steatohepatitis.

Gender has been described as an important factor in the outcome of various liver diseases including hepatic steatosis and inflammation [10], yet most studies only employed male mice (e.g. none of the above mentioned studies showed female data).

Hyperlipidemic APOE2ki mice are more prone to liver inflammation and reportedly a promising model for the progression of NAFLD to NASH [11,12]. The replacement of the mouse *Apoe *gene with the human *APOE2 *allele in APOE2ki mice decreases the hepatic uptake of (very) low-density lipoproteins and, thus, causes mixed hyperlipidemia [13]. In these studies female mice were more prone to inflammation, but it remains unknown if modulation of the fat source may influence the outcome.

In this study, we show that dietary cholesterol plays a determining, independent role in inflammation and that liver health is especially affected in female mice by dietary cholesterol. The fatty-acid saturation, on the other hand, plays only an insignificant role in terms of hepatic steatosis or inflammation.

## Methods

### Animals and treatments

C57BL/6J and *Apoe*^*tm(APOE2) *^mice on the C57BL/6J background were used. *Apoe*^*tm(APOE2) *^mice, in which the mouse *Apoe *gene is replaced by the human *APOE2 *gene [13], were kindly provided by P. van Gorp (Dept. of Molecular Genetics, Maastricht University). C57BL/6J and *Apoe*^*tm(APOE2) *^mice are designated as WT and APOE2ki, respectively, in text and figures. Both male and female mice were studied. Two mice per cage were kept in a temperature-controlled facility with fixed 12 h light-dark cycles and free access to food and water. Twice weekly, food intake and body weight were measured. After three weeks on the specified diet, mice were sacrificed in the morning to avoid chronobiological variation. The study was approved by the Committee for Animal Care and Use of Maastricht University.

Mice were anesthetized by intraperitoneal injection of 9 mL/kg body weight of a mixture of midazolam (Dormicum^®^, Roche, Almere, Netherlands), fluanisone/fentanyl citrate (Hypnorm^®^, VetaPharma, Leeds, UK), and sterile water (1:1:2 (v/v)). Fifteen minutes after injection, the mice were laparotomized to sample caval blood and tissues. The liver and epididymal/parametrial fat were weighed and snap-frozen for further analysis.

### Diets

**Figure 1:**
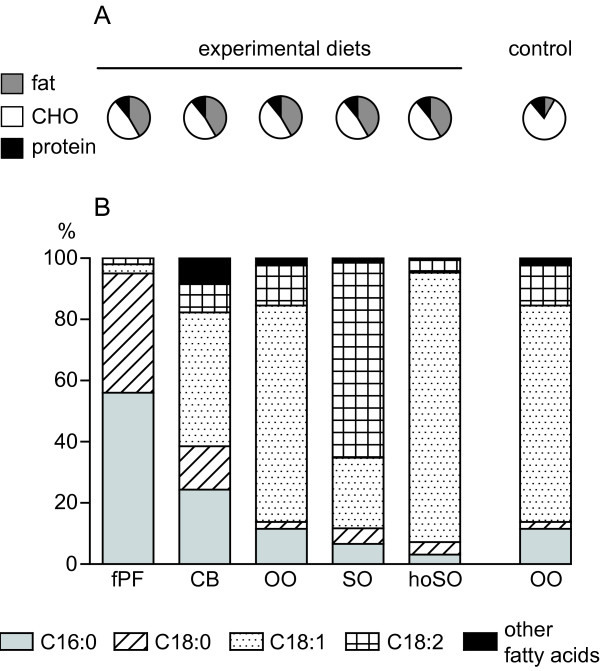
**Macronutrient (en%; panel A) and fatty-acid composition (panel B) of the diets**.

Two semi-synthetic low-fat diets (without and with supplemented cholesterol; 11 en% protein, 81 en% carbohydrates, and 8 en% fat; 3.8 kcal/g) and five semi-synthetic, high-fat diets with the same macro-nutrient composition (11 en% protein, 48 en% carbohydrates, and 42 en% fat including 0.2% (w/w) cholesterol; 4.6 kcal/g) and were purchased from Research Diets Inc. (New Brunswick, NJ, USA) and stored at 4ºC until use. High-fat diets differed only in the source of fat and saturation of the fatty acids (Figure 1 and additional file 1, table S1: Composition of control and high-fat diets). In addition to the commonly used cocoa butter, olive and sunflower oil, we included a sunflower-oil diet (hoSO) with a very high oleic-acid content (~88%; Trisun^®^) and a highly saturated, fractionated palm-fat (fPF) diet with high palmitic acid content (~ 56%; Optifat^®^).

### Oil-red O quantification

Fat accumulation in the livers was visualized using Oil-red O staining. Five random high-power photographs (20 × objective) were recorded per liver and quantified with the Qwin™ image analysis software (Leica Microsystems B.V., Wetzlar, Germany). The total Oil-red O-positive surface (= volume) fraction and the fat droplet size distribution were determined. Macrosteatosis was defined as the volume percentage of fat globules with a radius larger than ~2 μm.

### Immunohistochemistry

Formalin-fixed sections were used to stain for the presence of myeloperoxidase (Mpo, a neutrophil marker). After deparaffinization, sections were blocked with 0.6% H_2_O_2 _in 80% methanol for 15 min. Non-specific binding was blocked with 10% normal goat serum (NGS) in Teng-T buffer (100 mM Tris-HCl, pH 8.0, 50 mM EDTA, 1.5 mM NaCl, 2.5% gelatin, 0.5% Tween) for 30 min. Liver sections were incubated with primary antibody (rabbit-α-Mpo (Dako, Glostrup, Denmark), 1:1500 in 10% NGS/TengT-buffer) for 1 h at room temperature, followed by secondary antibody (biotinylated goat-anti-rabbit, Dako) and ABC-HRP-complex for 30 min. Antibody binding was visualized with 3,3-diaminobenzidine (DAB; Sigma-Aldrich, MO, USA).

Frozen liver sections (7 μm) were stained for the presence of Cd68-positive cells (residential phagocytic macrophages) and Cd11b-positive cells (infiltrated/activated macrophages). Sections were air-dried and fixed in 100% ice-cold acetone. Nonspecific binding was blocked by pre-incubation in PBS containing 4% (v/v) fetal calf serum (FCS) and 240 μL/mL avidin D solution (Vector Laboratories, Burlingame, CA, USA). Cd68 (Rat-anti-Cd68, Hycult Biotechnology, Uden, The Netherlands) and Cd11b (Rat-anti-Cd11b, R&D Systems, McKinley, MN, USA) antibodies were dissolved in 4% (v/v) FCS/PBS with 240 μL/mL biotin D solution (Vector Laboratories) and incubated on the section for 1 h. The secondary antibody was preincubated with 4% (v/v) FCS/2% normal mouse serum (NMS)/PBS for at least 10 minutes to reduce nonspecific binding. Antibody binding was visualized with 3-amino-9-ethylcarbazole (AEC; Sigma-Aldrich). Sections were scored blinded and independently by three persons and scores are depicted as median and quartile values per group (definition of scoring grades, see additional file 2, table S2: Cd11b-scoring criteria).

### Plasma analysis

Whole-blood glucose was measured with a glucose meter (Ascencia Contour, Bayer, Leverkusen, Germany). Heparinized plasma was prepared from remaining blood, snap-frozen in liquid nitrogen and stored at -80°C. Plasma triglycerides were determined using the Triglyceride/Glycerol Blanked kit (Roche Diagnostics, Almere, Netherlands) and non-esterified fatty acids (NEFA) with the NEFA-C kit (WAKO Chemicals, Neus, Germany). Total plasma cholesterol levels were determined with an enzymatic assay (bioMérieux, Marcy l'Etoile, France). Lipoprotein profiles of pooled plasma samples were determined using the AKTA chromatography system equipped with a Superose 6PC 3.2/30 column (Amersham Biosciences, Diegem, Belgium) [11].

### Liver lipid analysis

Hepatic triglyceride content was analyzed as described previously [11]. Briefly, ~50 mg of frozen liver tissue was homogenized with an Ultraturrax and sonicated for 30 sec in 1.0 mL SET buffer (sucrose 250 mM, EDTA 2 mM and Tris-HCl 10 mM, pH 7.4). Complete cell destruction was achieved by one freeze-thaw cycle and two 15-sec cycles of sonication (amplitude: 10 μm). Liver triglycerides were analyzed using the same kit as described for plasma triglyceride analysis. Protein content was measured with the bicinchoninic acid (BCA) method (Pierce, Rockford, IL, USA). To determine the fatty-acid composition of the liver, the samples of every group were pooled, and fat was extracted and separated by thin layer chromatography (TLC) [14]. The triglyceride fraction was further analyzed by gas chromatography [15].

### Quantitative PCR

Total RNA was isolated from approx. 50 mg tissue using the TRI REAGENT™ (Sigma-Aldrich) and further purified by 2 M LiCl precipitation. cDNA synthesis was performed on denatured RNA (5 min at 65°C, then quenched on ice) with iScript™ cDNA-synthesis kit (Bio-Rad Laboratories, Veenendaal, The Netherlands). Real-time PCR was performed in the iQ5 thermal cycler (Bio-Rad Laboratories) and the corresponding PCR-reagents (iQ SYBR Green Supermix™), using specific primers for chemokine (C-C motif) ligand 2 (*Ccl2*), tumor necrosis factor α (*Tnf-α*) and macrosialin (*Cd68*) (for primer sequences, see additional file 3, table S3: Primer sequences for quantitative PCR). *18S *ribosomal RNA was used as a reference. Relative mRNA levels were quantified with the LinReg software [16], which uses linear regression of the time-dependent increase in Log(fluorescence).

### Statistical methods

Data are shown as means ± SEM. Comparisons were computed with SPSS version 15 (SPSS Inc., IL USA), using ANOVA and post-hoc analysis. For data with equal variance, the Hochberg's GT2 test was used, whereas for the remaining cases, Games-Howell was applied. P < 0.05 was considered significant.

## Results

### Animal characteristics

Biometric details (age and body weight at the start of the experiment, and glucose, fat pad, liver weight at the end of the experiment) of the mice in this study are given in additional file 4, table S4: Biometric details of experimental mice. Male WT mice on the cocoa-butter diet consumed more food (P < 0.02), but mice on all other diets had similar energy intakes per day during the second and third week (additional file 5, figure S1A: Energy intake and change in body weight of mice fed the respective high-fat diets). Furthermore, WT mice refused the highly saturated fPF diet. To test whether another background would respond differently, mice of the obesity-resistant FVB strain [17] were fed the fPF diet. Food intake of FVB mice on the fPF diet was normal and comparable to food intake of WT mice on the other diets. No significant differences in weight gain were seen between or within groups of mice subjected to a high-fat diet (additional file 5, figure S1B: Energy intake and change in body weight of mice fed the respective high-fat diets). An effect of gender on weight changes was only seen in the WT mice (P = 0.03). Male WT mice on high-fat diets show significantly increased plasma glucose concentrations compared to the low-fat diet (P < 0.001) and compared to female WT mice on high-fat diets (P = 0.001; additional file 4, table S4: Biometric details of experimental mice). In APOE2ki mice no significant differences were detectable between high- and low-fat diets or genders.

### Effect of cholesterol supplementation

**Figure 2:**
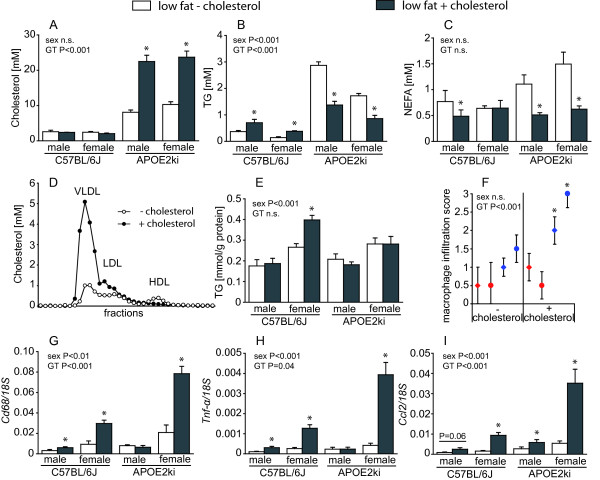
**Plasma and liver parameters of low-fat diets without or with supplemented cholesterol**. Plasma levels of cholesterol (panel A), triglycerides (panel B), and NEFA (panel C) in WT and APOE2ki mice fed the respective diets. Panel D depicts the FPLC analysis of plasma lipoproteins of male APOE2ki mice. The plasma lipoprotein profiles of female APOE2ki mice were similar of those in male mice. The concentration of enzymatically measured liver triglycerides is shown in panel E. Panel F represents the macrophage activation score on a scale of 0 to 3 (see additional file 2, table S2: Cd11b-scoring criteria) based on the presence of Cd11b-positive cells in the liver of the mice. Median values of all observations of each group are indicated by diamonds (male mice) and circles (female mice) (red: WT, blue: APOE2ki). The error bars represent the first and third quartiles of each group, *P < 0.05 between the diets. *Cd68 *mRNA expression is depicted in panel G, *Tnf-α *expression in panel H, and *Ccl2 *expression in panel I. Levels are normalized with 18S rRNA. Values in panels A, B, C, E, G, H, I are shown as means ± SEM of 6-10 mice per group, * P < 0.05.

The addition of 0.2% cholesterol to a low-fat diet did not affect plasma cholesterol levels in WT mice, but increased plasma triglycerides ~2-fold (P < 0.001) and, in males only, plasma NEFA levels decreased to ~75% (Figure 2A-C). Dietary cholesterol modulated the plasma lipid profile in APOE2ki mice significantly (Figure 2A-D). Plasma cholesterol increased ~2.5-fold when cholesterol was added to the diet (Figure 2A), whereas plasma triglycerides (Figure 2B) and non-esterified fatty acids (Figure 2C) decreased to ~50% (P < 0.001). A consistent gender difference was only found for plasma triglycerides (P < 0.001). FPLC analysis revealed that the addition of cholesterol to the diet increased VLDL and LDL concentrations, and decreased HDL concentration in APOE2ki mice (Figure 2D). The cholesterol-containing low-fat diet increased liver TG ~1.4-fold in female WT mice (P < 0.001; Figure 2E). Male WT and APOE2ki mice, however, showed no differences in liver triglyceride concentrations.

As shown in Figure 2F, APOE2ki mice showed more activated macrophage infiltration in the liver than WT mice on a cholesterol-free low-fat diet, which was yet further increased upon cholesterol addition (P < 0.001). RT-PCR of *Cd68*, *Tnf-α *and *Ccl2 *showed that especially female mice responded with an increased expression of these genes when cholesterol was present in the diet (Figure 2G-I; P = 0.003). Since Western-style diets usually contain a liberal amount of cholesterol, we continued our study with dietary fats with a different degree of saturation in the presence of 0.2% cholesterol.

### Plasma parameters are not modulated by the degree of saturation of the dietary fat

**Figure 3:**
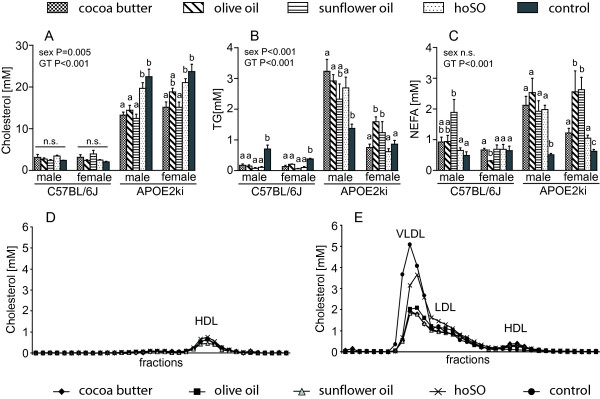
**Plasma parameters**. Plasma levels of cholesterol (panel A), triglycerides (panel B), and non-esterified fatty acid (NEFA; panel C) in WT and APOE2ki mice fed the respective diets. FPLC analysis of plasma lipoproteins of male WT (D) and APOE2ki (E) mice. The plasma lipoprotein profiles of female mice were similar to those of male mice. Values are shown as means ± SEM of 6-10 mice per group. Different letters indicate a significant difference (P < 0.05) between these groups. GT: genotype; n.s.: non significant.

The plasma cholesterol profile differed substantially between WT and APOE2ki mice (Figure 3A). The concentration of HDL did not differ between the different diets and genotypes (Figure 3D, E). In WT mice, neither LDL nor VLDL were found in plasma (Figure 3D). Plasma lipid profiles of APOE2ki mice on either the CB-, OO- or SO-diet were similar, but revealed lower VLDL concentrations than on both the hoSO- and the low-fat control diet (Figure 3E). No gender differences were found (data not shown).

WT mice showed no difference in total plasma cholesterol levels between the low- and high-fat diets, and between the different high-fat diets themselves (Figure 3A). On average, in APOE2ki mice, plasma cholesterol in mice fed the CB, OO or SO diets was ~60% of that on the low-fat diet (P = 0.001). Furthermore, APOE2ki mice had 5-10-fold higher plasma triglyceride and 2-fold higher non-esterified fatty acid levels than WT mice (P < 0.001 and P < 0.01, respectively; Figure 3B, C). APOE2ki mice on the low-fat diet show significantly reduced plasma TG and non-esterified fatty acids when compared to the high-fat diets (P < 0.001). Female APOE2ki mice had ~2-fold lower plasma triglyceride concentrations than male APOE2ki mice (P < 0.001). No significant effects were found between the different high-fat diets in WT or APOE2ki mice. Thus, the main differences in plasma lipid profiles were caused by the genotype and gender rather than the type of fat.

### Saturation of dietary fat influences neither the degree of steatosis nor the distribution of fat in the liver

**Figure 4:**
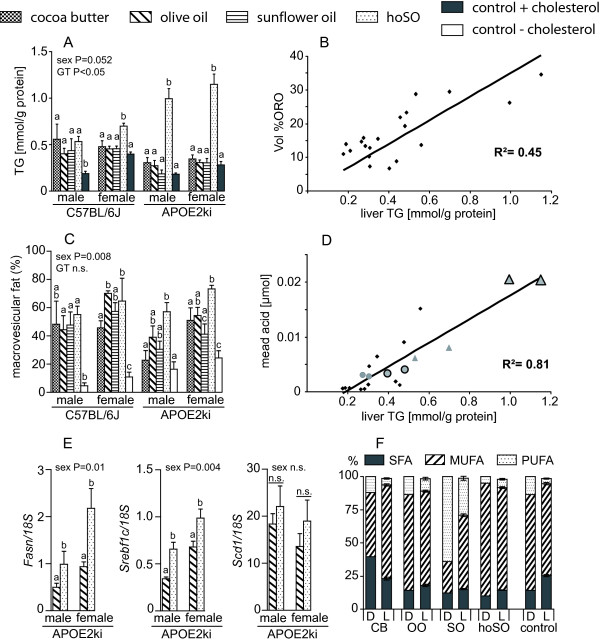
**The concentration of triglycerides in liver**. Panel A shows the triglyceride concentration measured enzymatically. Panel C indicates the percentage of fat present as >2 μm (macrovesicular) globules determined morphometrically in Oil-red O-stained sections. The correlation of the biochemical assay (Panel A) and the histochemical assay (Panel C) is shown in panel B (P < 0.001). Assuming ~150 mg protein per gram liver wet weight, 1 vol% ORO represents ~4 μmol TG/gram liver. Panel D: The concentration of mead acid in the liver TG fraction correlates positively with the hepatic triglyceride concentration. The grey triangles indicate hoSO- and the grey circles OO-fed mice, with the APOE2ki mice emphasized with a black contour; all other small black dots indicate the other diet groups. Note the very high mead acid concentrations in hoSO-fed APOE2ki compared to WT mice. Also note that livers with a low fat content have no detectable mead acid levels. Panel E: *Fasn, Srebf1*, and *Scd1 *mRNA expression in the liver of hoSO and olive oil-fed APOE2ki mice. Panel F compares the composition of liver fat as determined by gas chromatography and the dietary fatty acid composition (D: fatty-acid composition in diet; L: fatty-acid composition in liver triglycerides). Values are indicated as means ± SEM. Different letters indicate a significant difference (P < 0.05) within each group. GT: genotype; n.s.: non significant.

We investigated lipid accumulation in the liver and its distribution in macro- and microvesicular fat globules (Figure 4). In WT mice, the feeding of any of the high-fat diets resulted in higher fat content in liver compared to the low-fat diet (male: ~2-fold, P < 0.05; female: ~1.2-fold, P = n.s.; Figure 4A). APOE2ki mice showed no extra fat accumulation in the liver when fed a CB, OO or SO high-fat diet. Consequently, WT mice had significantly more fat in their livers than APOE2ki mice when fed a high-fat diet (P < 0.007). The quantification of total liver fat with Oil-red O confirmed these results. A direct comparison showed that the biochemical and histochemical assays of fat accumulation in the liver correlated well (Figure 4B). Quantitative morphological analysis revealed a continuous size distribution of the fat vesicles, regardless of the diet consumed. The size distribution of these vesicles changed towards a larger diameter in animals on a high-fat diet (Figure 4C), demonstrating that diet-induced steatosis is macrovesicular.

The hoSO diet differed strikingly in its effects from the other fats, in particular in APOE2ki mice. These mice showed a remarkable ~4-fold increase in liver fat relative to the other high-fat diets (P < 0.001; Figure 4A). Furthermore, mead acid (Figure 4D) and mRNA levels of fatty acid synthase (*Fasn*) and sterol regulatory element-binding transcription factor 1 (*Srebf1*), both markers of increased lipogenesis (Figure 4E), were significantly upregulated compared to APOE2ki mice fed a high-fat OO diet (P = 0.035 and P = 0.008, respectively). Stearoyl-CoA desaturase 1 (*Scd1*) mRNA was unchanged (Figure 4E).

### Saturation of dietary fat has only a minor effect on liver fat composition

The composition of liver fat of mice fed fats with different degrees of saturation showed a remarkable similarity (Figure 4F, additional file 6, table S5: Triglyceride fatty acid composition (mol%)). For all diets, oleic acid was the main fatty acid in the liver (~70%). Only the sunflower-oil diet was associated with a slightly, but consistently higher C18:2 content. As depicted in Figure 4F, the composition of the diet was therefore only partially reflected in the fat composition of the liver. Fatty-acid composition of SO-fed mice stands out with an increased very long chain fatty acids (>C22) of both the n-3 (22:5n-3, 22:6n-3; P < 0.001) and n-6 (20:4n-6, 22:4n-6; P < 0.001) series. On the other hand, n-3-to-n-6 ratio was significantly decreased in SO-fed animals due to the higher content of elongated n-6 PUFAs and lowering the dietary 18:3n-3 fatty acid compared with the other high fat diets. The liver of hoSO-fed mice, finally, contained more mead acid (P < 0.05) and showed a consistent trend for less elongation products (20:3n-6, 20:4n-6, 22:4n-6 and 22:5n-6; P < 0.07) than the liver of OO-fed mice (additional file 6, table S5: Triglyceride fatty acid composition (mol%)).

### Saturation of dietary fat does not influence liver inflammation

**Figure 5:**
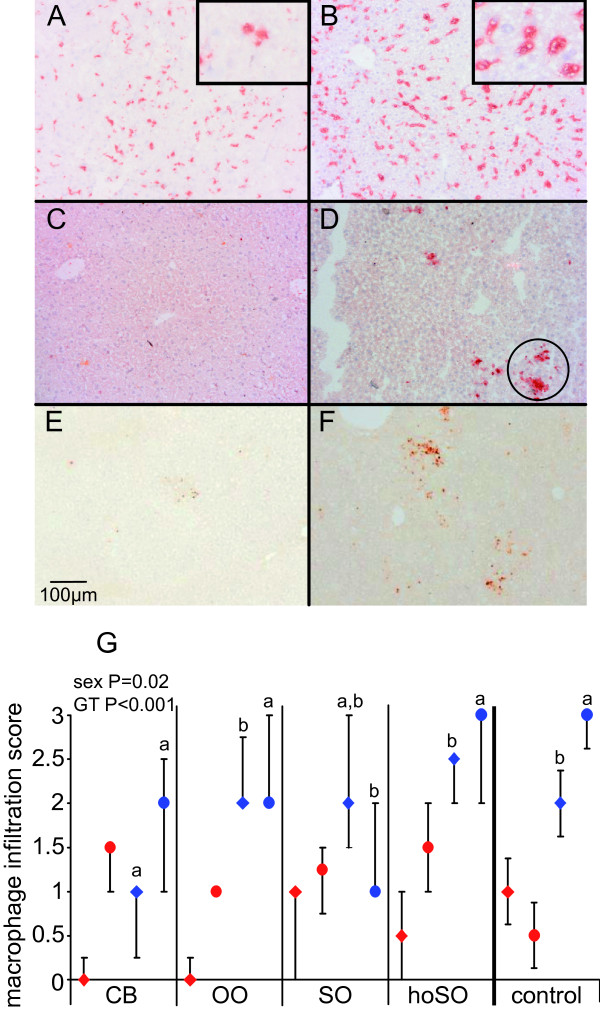
**Presence and distribution of macrophages and neutrophils in the liver**. Panels A and B: Cd68-positive macrophages in APOE2ki mice on the low-fat OO diet without cholesterol (A) or on high-fat OO diet with cholesterol (B). Insets show high-power magnifications of stained macrophages. Panels C and D: Cd11b-positive macrophages in a WT male liver (C; no macrophage activation) and a male APOE2ki liver (D; grade-2 macrophage activation) on the high-fat olive oil diet. Macrophage activation based on the presence of Cd11b-positive cells in the livers of animals of the respective experimental and control groups was graded on a scale of 0 to 3 (see additional file 2, table S2: Cd11b-scoring criteria). The circle indentifies a cluster of activated macrophages. Panels E and F: Mpo-positive cells in livers assigned a grade 3 for Cd11b-positive macrophages (E: without Mpo-positive cells; F: with Mpo-positive cells). Panel G: Median values of macrophage activation in each of the groups are indicated by diamonds (male) and circles (female mice) (red: WT, blue: APOE2ki). The error bars represent the first and third quartiles of each group. When not drawn, a quartile coincides with the median. Bars: 100 μm. Different letters indicate a significant difference (P < 0.05) between the diets within male or female APOE2ki mice. GT: genotype; n.s.: non significant.

**Figure 6:**
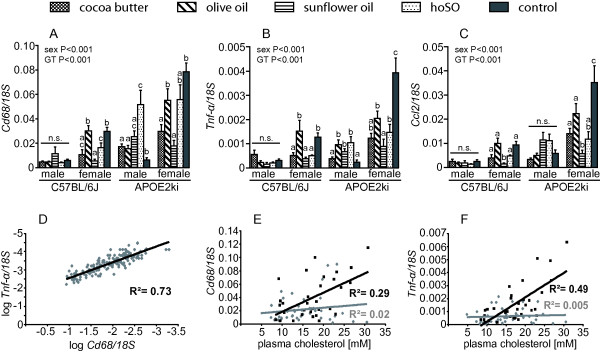
**Expression of inflammatory genes in livers of mice fed the respective high-fat diets**. *Cd68 *mRNA expression is depicted in panel A, *Tnf-α *mRNA expression in panel B, and *Ccl2 *mRNA expression in panel C. Levels are normalized with 18S rRNA concentration and expressed as means ± SEM. Different letters indicate a significant difference (P < 0.05) within each group. GT: genotype; n.s.: non significant. In panel D the expression of *Tnf-α *is correlated with *Cd68 *expression; in panels E and F, the expressions of *Cd68 *and *Tnf-α *are correlated with plasma cholesterol (grey: male, black; female), respectively. Note that the positive correlations in panels E and F are only seen in female mice.

Cd68-positive (resident) macrophages were abundantly present in the livers of all mice, with no difference between genotypes, gender, or diets. APOE2ki mice showed swollen Cd68-positive cells both on the high-fat and the low-fat diet containing cholesterol, but not on the low-fat diet without cholesterol (inserts of Figure 5A and 5B). Activated (Cd11b-positive) macrophages were, on the other hand, rarely present in the liver of WT mice (Figure 5C), but abundant in that of APOE2ki mice (P < 0.001; Figure 5D). Since no diet-dependent effects were detected, we conclude that macrophage activation was determined primarily by genotype (P = 0.001) and, to a lesser extent, by gender (Figure 5G). Mpo-positive cells (Figure 5E, F) were seen almost exclusively in livers with the highest grade of Cd11b-positive cells (24% of all samples with score 3 of APOE2ki mice). The higher inflammatory status of the APOE2ki mice was confirmed by real-time PCR. *Cd68*, *Tnf*-α, and *Ccl2 *mRNA contents were 2-6-fold higher in APOE2ki than in WT mouse livers (P < 0.001; Figure 6A, B, and 6C, respectively). In WT mice, we did not find a consistent induction of *Cd68*, *Tnf*-α, or *Ccl2 *mRNA expression by any of the diets.

There were subtle, but consistent gender differences in inflammatory response. Female mice showed a significantly higher density of Cd11b-positive cells and a higher expression of inflammatory markers. Interestingly, liver inflammation remained high in female mice on the low-fat control diet, whereas the number of Cd11b-positive cells and inflammatory gene expression decreased in male mice. OO-fed female mice showed a consistently higher inflammatory gene expression, whereas SO oil caused a lower inflammatory response (fewer Cd11b-positive cells and less *Cd68*, *Tnf*-α, or *Ccl2 *mRNA, P < 0.025 and P < 0.05, respectively) in female but not male mice.

### Plasma cholesterol and inflammation

Further analysis of the APOE2ki livers showed that *Cd68*, *Tnf*-*α *or *Ccl2 *mRNA expression were significantly correlated (R^2 ^= 0.73; P < 0.001; Figure 6D), indicating that all these markers reflect the inflammatory status of the liver. Further analysis revealed that plasma cholesterol levels correlated with *Cd68 *and *Tnf*-*α *mRNA expression in female mice (R^2 ^= 0.29 and 0.49, respectively), but were absent in male mice (R^2^<0.02; Figure 6E and 6F). In addition, there was a stronger correlation between plasma cholesterol and the degree of the accumulation of Cd11b-positive cells in female mice (R^2 ^= 0.31; P = 0.001) than in male mice (R^2 ^= 0.15; P = 0.013; additional file 7, figure S2: Correlation of liver Cd11b scoring with plasma cholesterol levels).

## Discussion

The experimental protocol of our study allowed a comparison of the effects of dietary fat saturation index in two mouse models which differ in their susceptibility to steatosis and steatohepatitis. C57BL/6J mice are widely used in dietary fat studies, while APOE2ki mice on the C57BL/6J background are a humanized mouse model for hyperlipidemia. Surprisingly, the choice of fat plays only a minor role in liver steatosis and inflammation. Instead, cholesterol significantly modulates plasma and liver values, while hepatic inflammation is also modified by the female gender. Furthermore, we found evidence that a relative deficiency of essential fatty acids may affect hepatic fat metabolism. Finally, our findings show a clear effect of gender, so female mice should be included in further studies of NAFLD.

### Saturation of the dietary fats does not affect steatosis and liver inflammation

All fats were from plant sources. The fPF, which is used in animal feed, would have been ideal to reveal the roles of a high palmitate (~55%) content and a high degree of saturation (~95%). Dietary palmitic acid is partly metabolized to ceramides, which may cause inflammation [18]. A high-fat diet with only 30% of fPF (12% less than used in this study) was reported to be tolerated by WT mice [19]. Based on this report and the fact that FVB-mice accepted the fPF-based diet, we conclude that fPF is not toxic, but poorly tolerated by WT mice. Since high-fat diets based on palm oil (~45% palmitate and ~50% saturation) are well tolerated by WT mice [20], the melting point of the fat (fPF ~53°C versus CB ~35°C) rather than the palmitic acid content appears to determine tolerance. Furthermore, cocoa butter, the diet with the highest palmitic acid (~24%) content and highest degree of saturation (~65%) in this study, was also very well tolerated and even overconsumed by male WT males.

The degree of saturation of the dietary fat did not affect steatosis or liver inflammation, which appears to contradict the health claims for e.g. olive oil [21]. We used refined grade oils and fats, so that the discrepancy may be attributable to secondary compounds, like waxes and polyphenols [22]. However, olive oil did also not ameliorate steatosis or liver inflammation in a long-term (12 weeks) high-fat diet study in rats [23]. Relative to other fats, olive oil diminished steatosis in mice fed a methionine-choline deficient (MCD) diet, but aggravated inflammation [24]. These latter results have to be interpreted with care, though, since an MCD diet itself already induces a disturbance of fatty-acid metabolism [24].

The discrepancy between the effects of olive oil (~72% oleic acid content) and hoSO (~90% oleic acid) on liver fat content of APOE2ki mice was unexpected. The hoSO differs from all other dietary fats tested in its content of both saturated and polyunsaturated fatty acids (<50% of that in the other diets). We suspect that especially the low dietary PUFA content (~5% versus 14% in OO) may cause disturbances in lipid metabolism, because feeding mice a diet deficient in 18:2 and 18:3 fatty acids for eight weeks causes hepatic steatosis [25]. If insufficient essential fatty acids are present in the diet, oleic acid rather than linoleic or α-linolenic acid is elongated and desaturated to form mead acid (20:3n-9) by Δ6-desaturase [26]. In agreement with this hypothesis, we found that hoSO-fed mice have a 1.6-fold increased mead acid content in their livers. We believe that APOE2ki mice were especially susceptible, because the absolute influx of fat into the liver is lower than in WT mice. Upregulation of lipogenesis in the hoSO-fed APOE2ki mice is supported by the ~2-fold higher *Fasn *and *Srebf1 *mRNA levels when compared to olive oil-fed mice. However, palmitic and palmitoleic acid concentrations, which must be synthesized *de novo*, since they are not present in the hoSO diet, remained unchanged compared to OO-fed mice.

### The size of hepatic fat vesicles correlates with dietary fat content and not with inflammation

If the majority of fat droplets that are visible in formaldehyde-fixed and paraffin-embedded sections is smaller or bigger than the nucleus of a hepatocyte, this liver is said to be micro- or macro-steatotic, respectively [27,28]. Microvesicular steatosis corresponds with a more serious inflammatory state [29]. Our finding is that all high-fat diets increased the diameter of the fat vesicles to a similar extent in WT and APOE2ki livers, even though the fat content of APOE2ki livers was not increased. This shows that the size distribution of fat vesicles in cryosections stained with Oil-Red O reflects dietary rather than hepatic fat content. These observations concur with human studies that nutritional fat excess induces macrovesicular steatosis [30-32].

### APOE2ki mice are protected against high-fat diet induced steatosis

Liver fat accumulation in WT mice on a high-fat diet was approximately twice that in APOE2ki mice, which, with the exception of the hoSO diet, did not accumulate extra fat. As in other hyperlipidemic mouse models (*Apoe*-ko, *Ldlr*-ko), the high plasma cholesterol and triglyceride levels reflect the reduced uptake of apolipoprotein particles into the liver. For that reason, these mouse models are less likely to develop liver steatosis [33]. However, APOE2ki mice on a high-fat diet enriched in short- and medium-chain fatty acids (<C14), such as milk, do develop fatty livers [11], because these fatty acids are not incorporated into chylomicrons [34].

### Different dietary fats mainly accumulate as oleic acid in the liver

An important finding of our study was that the composition of dietary fat has little influence on the chemical composition of stored liver fat. Liver fat composition was similar for all diets, with oleic acid as its main constituent. This finding demonstrates that liver fat composition is determined by liver metabolism rather than dietary fat composition. The conversion of liver fats to monounsaturated fats by stearoyl-CoA desaturase (Scd-1) may avoid accumulation of saturated fats, which results in inflammation and apoptosis of hepatocytes [35]. Even sunflower oil-fed mice preferentially stored monounsaturated triglycerides, suggesting oxidation of PUFAs and recycling of their carbons for *de-novo *fatty-acid synthesis [36,37]. Some of the linoleic acid and α-linolenic acid in the sunflower oil underwent further elongation and desaturation, as can be deduced from increase n-3 (22:5n-3, 22:6n-3) and n-6 (20:4n-6, 22:4n-6) fatty acid series in liver fat. In agreement with previous studies these endogenously synthesized PUFAs were not able to increase β-oxidation via PPARα activation or suppress lipogenesis via Srebp1c as observed with purified fish oil supplements (22:5n-3, 22:6n-3) [38].

### Gender effect in NAFLD

Female APOE2ki mice showed a higher inflammatory response than male APOE2ki mice which may be due to differences in susceptibility of Kupffer cell activation (see section on liver inflammation). Female mice are more prone to develop endotoxin-induced inflammation due to increased *Tnf-α *expression by Kupffer cells [39]. Furthermore, female TLR4^-/- ^mice have a more attenuated inflammatory response upon a high-fat diet than WT littermates. This effect was blunted in male mice, indicating that females have a more sensitive TLR4-mediated inflammatory response [40]. In female, as opposed to male APOE2ki mice, plasma cholesterol correlated with inflammatory markers. Kupffer cells of APOE2ki mice take up cholesterol from plasma [41]. Progesterone blocks the postlysosomal transport of cholesterol in female mice [42,43]. As a consequence, cholesterol can not be exported from the Kupffer cells and inflammation is sustained. Female NASH-patients may, therefore, benefit more from cholesterol-lowering treatments than males.

We have shown that female mice, irrespective of genotype, display consistently a lower inflammatory status on the SO diet. With 25 en% of linoleic acid and only 0.2 en% of α-linolenic acid, SO has a much higher pro-inflammatory linoleic acid (n-6) to anti-inflammatory α-linolenic acid (n-3) ratio than the other diets. All SO-fed mice (genotypes and genders) had a lower Δ5-desaturation index of n-6 fatty acids (the ratio of 20:4(n-6) over 20:3(n-6) fats) in the liver than mice fed the other high-fat diets. Female mice must, therefore, have a thus far unknown, essential fatty-acid-independent mechanism to decrease inflammation.

We report here that female WT mice on a low-fat diet accumulate liver triglycerides to a same extent as on high fat diets. PPARα is less active in female than in male mice [44]. In addition, high carbohydrate levels in the low-fat diet stimulate *de-novo *lipogenesis, as indicated by the ~2 fold increased *Srebf1 *and ~5 fold increase in *Fasn *expression (data not shown). This combination can explain the higher liver triglyceride levels in the low-fat diet. When cholesterol was omitted from the low-fat diet, liver triglycerides in female WT mice decreased significantly for an unknown reason that must be linked to dietary cholesterol.

### Factors contributing to liver inflammation

The number of Cd68-positive resident macrophages [45] did not differ between diets and genotypes, but, as observed previously [11,41], macrophages were more swollen in the livers of APOE2ki mice fed a cholesterol-containing diet than a diet without cholesterol. Apparently, these macrophages take up excess cholesterol from the hyperlipidemic plasma. The presence of Cd11b-positive, newly recruited macrophages in liver was higher in APOE2ki than in WT mice [45], but similar for all cholesterol-supplemented diets. The accumulation of cholesterol in liver macrophages of APOE2ki mice probably induces recruitment of activated macrophages. The significant correlation between plasma cholesterol levels and the expression of the inflammatory markers Cd11b, *Cd68*, and *Tnf-*α in liver supports this reasoning, albeit that the contribution of plasma cholesterol to the inflammatory state is most apparent in female mice. This suggests that modulation of dietary cholesterol might be especially beneficial for females. Nevertheless, myeloperoxidase (Mpo)-positive cells were recruited to the liver in 11% of all APOE2ki mice and, specifically, to 25% of the livers with grade 3 of CD11b-inflammation. Infiltration of Mpo-positive cells did not correlate with a specific dietary fat. Since the infiltration of Mpo-positive cells is a feature of human NASH [46,47], APOE2ki mice are a promising animal model for NASH. Liver triglyceride content did not correlate with macrophage activation or neutrophil infiltration, arguing against the accumulation of fat in hepatocytes as the trigger of macrophage activation, as implied by the two-hit hypothesis [2].

## Conclusions

Our data emphasize the important role of dietary cholesterol in the development of steatosis and steatohepatitis. Dietary cholesterol regulates plasma cholesterol, triglyceride, and NEFA concentrations. Plasma cholesterol, in turn, correlates very tightly with liver inflammation in female mice. Saturation of dietary fatty acids, on the other hand, affects the severity of NAFLD only to a limited extent. We observed that oleic acid is the main fatty acid component in liver, irrespective of the dietary fat source. This study will serve as an excellent reference for future studies on effects of macronutrients, since it covers the whole spectrum of fatty acid saturation. Furthermore, our findings demonstrate the necessity of gender-specific research in the field of the metabolic syndrome.

## List of abbreviations

APOE2ki: human apolipoprotein E2 knock-in; CB: cocoa butter; Cd11b: integrin alpha M (Itgam); Ccl2: chemokine (C-C motif) ligand 2 a.k.a.; (Mcp-1): monocyte chemoattractant protein-1; FPLC: fast protein liquid chromatography; fPF: fractionated palm fat; HDL: high-density lipoprotein; hoSO: high oleic acid sunflower oil; LDL: low-density lipoprotein; LPS: lipopolysaccharide; MUFA: monounsaturated fatty acid; NAFLD: non-alcoholic fatty liver disease; NASH: non-alcoholic steatohepatitis; NEFA: non-esterified fatty acid; OO: olive oil; PUFA: polyunsaturated fatty acid; SO: sunflower oil; TG: triglycerides; Tnf-α: tumor necrosis factor α; VLDL: very low-density lipoprotein;

## Competing interests

The authors declare that they have no competing interests.

## Authors' contributions

TMC and SCGC performed all experiments, analyzed the data and wrote the manuscript. CHCD, WHL, and SEK designed research and had primary responsibility for final content. All authors read and approved the final manuscript.

## Supplementary Material

Additional file 1 **Composition of control and high-fat diets**. The composition of the fatty acids of the different diets with respect to saturation is represented in a table. Opens with Adobe Acrobat Reader. Click here for file

Additional file 2 **Cd11b-scoring criteria**. The criteria for the scoring of Cd11b-staining are shown. Opens with Adobe Acrobat Reader. Click here for file

Additional file 3 **Primer sequences for quantitative PCR**. Sequences of primers used for quantitative PCR. Opens with Adobe Acrobat Reader. Click here for file

Additional file 4 **Biometric details of experimental mice**. Age, body weight, blood glucose concentration, fat pad and liver weight of the mice are shown. Opens with Adobe Acrobat Reader. Click here for file

Additional file 5 **Energy intake and change in body weight of mice fed the respective high-fat diets**. The values present the average energy intake (Panel A) and weight change (Panel B), respectively, per mouse per day in the second and third week of the experimental diet. Data are expressed as means ± SEM of 6-10 mice per group. Opens with Adobe Acrobat Reader. Click here for file

Additional file 6 **Triglyceride fatty acid composition (mol%)**. Fatty acid composition (mol%) of liver triglycerides. Opens with Adobe Acrobat Reader. Click here for file

Additional file 7 **Correlation of liver Cd11b scoring with plasma cholesterol levels**. The correlation of liver Cd11b scoring with plasma cholesterol levels depicted in grey are the males, and in black are the females. Opens with Adobe Acrobat Reader. Click here for file
