# Supplementary material for: Dietary cholesterol, female gender and n-3 fatty acid deficiency are more important factors in the development of non-alcoholic fatty liver disease than the saturation index of the fat
Source: Nutr Metab (Lond). 2011 Jan 24;8:4. doi: 10.1186/1743-7075-8-4 (PMC3045875; doi:10.1186/1743-7075-8-4)
Supplement: Additional file 1 — Composition of control and high-fat diets. The composition of the fatty acids of the different diets with respect to saturation is represented in a table. Opens with Adobe Acrobat Reader. [file 1743-7075-8-4-S1.PDF]

Additional table 1 - Composition of control and high-fat diets. The composition of the fatty acids in the respective diets with respect to saturation is shown. The high-fat diets contain 42 en% fat, whereas the control diet ("Control") contains 8 en% olive oil.

%	Fractionated palm fat	Cocoa butter	Olive oil	Sunflower oil	High oleic acid sunflower oil	Control (with or without cholesterol)
Saturated (SFA)	95	39.6	14.2	12.2	7.2	14.2
Monounsaturated (MUFA)	3	48.4	72.1	23.7	88.1	72.1
Polyunsaturated (PUFA)	2	12.0	13.6	64.2	4.6	13.6
Carbohydrates (en%)	48	48	48	48	48	81
Protein (en%)	11	11	11	11	11	11
Choline (%)	0.2	0.2	0.2	0.2	0.2	0.2
Cholesterol (%)	0.2	0.2	0.2	0.2	0.2	0.2 or 0
