# Supplementary material for: Dietary cholesterol, female gender and n-3 fatty acid deficiency are more important factors in the development of non-alcoholic fatty liver disease than the saturation index of the fat
Source: Nutr Metab (Lond). 2011 Jan 24;8:4. doi: 10.1186/1743-7075-8-4 (PMC3045875; doi:10.1186/1743-7075-8-4)
Supplement: Additional file 2 — Cd11b-scoring criteria. The criteria for the scoring of Cd11b-staining are shown. Opens with Adobe Acrobat Reader. [file 1743-7075-8-4-S2.PDF]

Additional table 2 - Cd11b-scoring criteria. The scores are expressed per surface area (±400 mm^2)

Score	Number (N) and distribution (foci) of Cd11b-positive cells per image
0	N < 10 positive single cells; no foci
1	10 < N < 100 positive single cells; no foci
2	N > 100 positive cells, *or* foci
3	N > 100 positive cells *and* foci
