# Supplementary material for: Dietary cholesterol, female gender and n-3 fatty acid deficiency are more important factors in the development of non-alcoholic fatty liver disease than the saturation index of the fat
Source: Nutr Metab (Lond). 2011 Jan 24;8:4. doi: 10.1186/1743-7075-8-4 (PMC3045875; doi:10.1186/1743-7075-8-4)
Supplement: Additional file 3 — Primer sequences for quantitative PCR. Sequences of primers used for quantitative PCR. Opens with Adobe Acrobat Reader. [file 1743-7075-8-4-S3.PDF]

**Additional table 3 - Primer sequences for quantitative PCR**

| Name | 5'-3' forward | 5'-3' reverse | NCBI Accession nr. |
|---|---|---|---|
| *Ccl2* | GCTGGAGAGCTACAAGAGGATCA | ACAGACCTCTCTCTTGAGCTTGGT | NM_011333.3 |
| *Cd68* | TGACCTGCTCTCTCTAAGGCTACA | TCACGGTTGCAAGAGAAACATG | NM_009853.1 |
| *Tnf-α* | CATCTTCTCAAAATTCGAGTGACAA | TGGGAGTAGACAAGGTACAACCC | NM_013696.2 |
| *Fasn* | GATATTGTCGCTCTGAGGCTGTTG | GGAATGTTACACCTTGCTCCTTGC | NM_007988.3 |
| *Srebf1* | CCGGCTATTCCGTGAACATC | ATCCAAGGGCATCTGAGAACTC | NM_011480.3 |
| *Scd1* | GGCCTGTACGGGATCATACTG | GGTCATGTAGTAGAAAATCCCGAAGA | NM_009127.4 |
| *18S* | AGTTAGCATGCCAGAGTCTCG | TGCATGGCCGTTCTTAGTTG | NR_003278.1 |
