# Supplementary material for: Dietary cholesterol, female gender and n-3 fatty acid deficiency are more important factors in the development of non-alcoholic fatty liver disease than the saturation index of the fat
Source: Nutr Metab (Lond). 2011 Jan 24;8:4. doi: 10.1186/1743-7075-8-4 (PMC3045875; doi:10.1186/1743-7075-8-4)
Supplement: Additional file 4 — Biometric details of experimental mice. Age, body weight, blood glucose concentration, fat pad and liver weight of the mice are shown. Opens with Adobe Acrobat Reader. [file 1743-7075-8-4-S4.PDF]

Additional table 4 - Biometric details of experimental mice. Age and body weight were determined at the start of the experiment, and blood glucose concentration, fat pad and liver weight at sacrifice after 3 weeks of dietary intervention.

C57BL/6J, male (N=39)

	CB (N=5)	OO (N=5)	SO (N=5)	hoSO (N=7)	Control (N=10)	Control w/o chol (N=7)
age (weeks)	9.0±0.0	8.4±0.2	8.4±0.2	11.1±0.1	12.5±0.3	10.3±0.3
body weight (g)	25.4±1.0	22.3±1.0	25.0±0.7	25.5±0.4	24.7±0.3	26.4±0.8
blood glucose (mM)	14.4±1.2	17.5±0.4	12.2±1.0	10.3±0.8	9.4±0.5	8.9±0.9
fat pad (g)	0.36±0.03	0.46±0.08	0.59±0.08	0.45±0.03	0.36±0.02	0.41±0.04
liver weight (g)	1.27±0.10	1.18±0.04	1.25±0.04	1.43±0.04	1.40±0.03	1.28±0.07

C57BL/6J, female (N=38)

	CB (N=5)	OO (N=5)	SO (N=5)	hoSO (N=7)	Control (N=10)	Control w/o chol (N=7)
age (weeks)	11.0±0.0	12.0±1.0	11.6±0.4	12.0±1.0	12.4±0.4	11.0±1.0
body weight (g)	19.8±0.3	21.0±0.3	20.1±0.4	19.8±0.6	20.0±0.4	19.9±0.5
blood glucose (mM)	10.2±0.4	10.9±1.1	9.1±1.4	9.9±1.2	10.4±0.6	8.3±1.1
fat pad (g)	0.22±0.01	0.26±0.03	0.34±0.05	0.20±0.03	0.27±0.04	0.21±0.01
liver weight (g)	0.98±0.03	1.20±0.03	1.06±0.04	1.19±0.03	1.22±0.04	1.19±0.04

APOE2ki, male (N=50)

	CB (N=5)	OO (N=5)	SO (N=5)	hoSO (N=7)	Control (N=8)	Control w/o chol (N=7)
age (weeks)	13.2±0.4	12.8±0.3	12.9±0.3	13.0±1.5	13.2±0.8	14.3±1.1
body weight (g)	25.6±0.8	25.4±0.7	26.2±0.7	23.6±0.8	24.2±0.6	25.4±0.7
blood glucose (mM)	9.3±0.5	9.0±0.6	8.4±0.7	7.7±0.5	7.8±0.5	8.3±0.7
fat pad (g)	0.49±0.06	0.37±0.05	0.34±0.04	0.39±0.03	0.33±0.02	0.28±0.02
liver weight (g)	1.21±0.04	1.35±0.07	1.27±0.06	1.26±0.03	1.41±0.06	1.29±0.08

APOE2ki, female (N=48)

	CB (N=5)	OO (N=5)	SO (N=5)	hoSO (N=7)	Control (N=8)	Control w/o chol (N=7)
age (weeks)	12.8±0.2	12.7±0.2	12.9±0.3	13.3±1.5	12.8±0.5	14.1±0.6
body weight (g)	20.7±0.5	19.4±0.8	20.6±0.6	21.6±0.1	19.8±0.6	20.7±0.4
blood glucose (mM)	8.2±0.6	8.9±1.1	9.0±0.6	7.8±0.6	7.4±0.5	8.3±0.6
fat pad (g)	0.23±0.04	0.16±0.03	0.21±0.02	0.22±0.03	0.21±0.03	0.22±0.02
liver weight (g)	1.07±0.04	1.14±0.03	0.91±0.09	1.17±0.07	1.33±0.05	1.18±0.07
