# Supplementary figures and images for: Dietary cholesterol, female gender and n-3 fatty acid deficiency are more important factors in the development of non-alcoholic fatty liver disease than the saturation index of the fat
Source: Nutr Metab (Lond). 2011 Jan 24;8:4. doi: 10.1186/1743-7075-8-4 (PMC3045875; doi:10.1186/1743-7075-8-4)

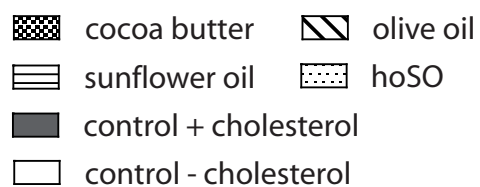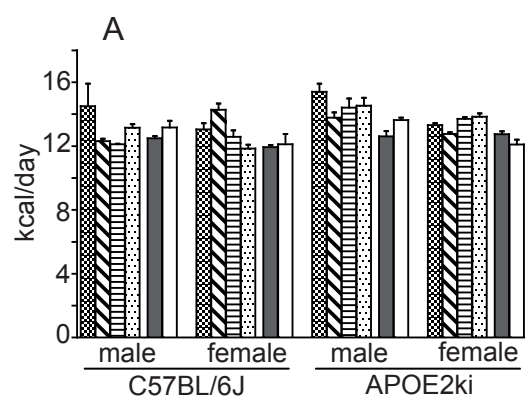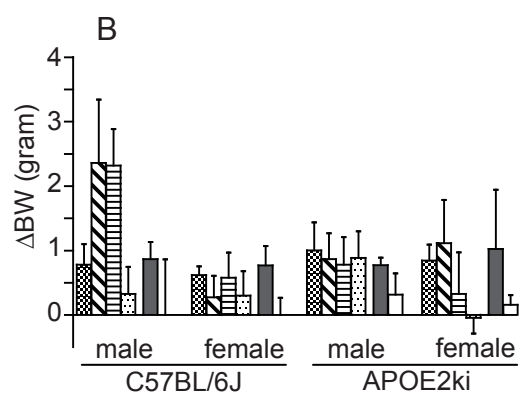

Supplement: Additional file 5 — Energy intake and change in body weight of mice fed the respective high-fat diets. The values present the average energy intake (Panel A) and weight change (Panel B), respectively, per mouse per day in the second and third week of the experimental diet. Data are expressed as means ± SEM of 6-10 mice per group. Opens with Adobe Acrobat Reader. [file 1743-7075-8-4-S5.PDF]

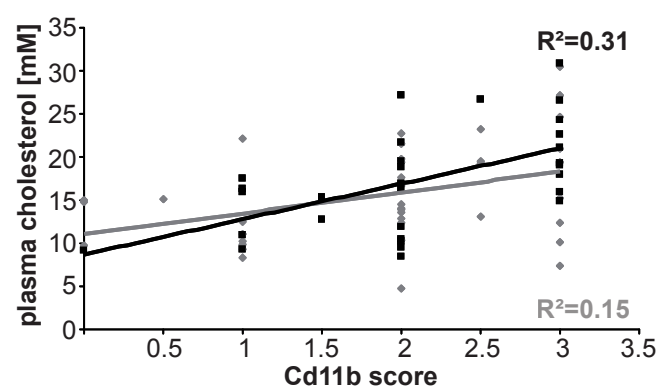

Supplement: Additional file 7 — Correlation of liver Cd11b scoring with plasma cholesterol levels. The correlation of liver Cd11b scoring with plasma cholesterol levels depicted in grey are the males, and in black are the females. Opens with Adobe Acrobat Reader. [file 1743-7075-8-4-S7.PDF]
