# Supplementary material for: Dietary cholesterol, female gender and n-3 fatty acid deficiency are more important factors in the development of non-alcoholic fatty liver disease than the saturation index of the fat
Source: Nutr Metab (Lond). 2011 Jan 24;8:4. doi: 10.1186/1743-7075-8-4 (PMC3045875; doi:10.1186/1743-7075-8-4)
Supplement: Additional file 6 — Triglyceride fatty acid composition (mol%). Fatty acid composition (mol%) of liver triglycerides. Opens with Adobe Acrobat Reader. [file 1743-7075-8-4-S6.PDF]

Additional table 5 - Triglyceride fatty acid composition (mol%)

	Cocoa butter	Olive oil	Sunflower oil	Trisun oil	Control
Saturated					
14:0	0.41±0.04	0.43±0.03	0.35±0.02	0.41±0.02	0.57±0.02
16:0	19.55±0.92	15.60±0.75	12.88±0.39	12.39±0.25	22.63±0.78
18:0	2.63±0.26	1.35±0.08	1.55±0.14	1.16±0.08	1.85±0.20
20:0	0.18±0.07	0.17±0.08	0.20±0.06	0.13±0.05	0.11±0.05
21:0	0.14±0.02	0.06±0.01	0.02±0.01	0.10±0.01	0.05±0.03
22:0	0.01±0.01	0.14±0.11	0.01±0.01	0.02±0.01	0.00±0.00
24:0	0.00±0.00	0.05±0.02	0.02±0.02	0.01±0.01	0.00±0.00
Monounsaturated					
16:1(n-7)	2.24±0.27	1.82±0.19	1.07±0.14	1.24±0.11	4.04±0.39
18:1 tr*	0.11±0.01	0.12±0.02	0.06±0.02	0.08±0.01	0.21±0.03
18:1(n-9)	62.62±1.68	64.25±1.17	50.99±1.13	71.72±1.33	56.47±1.57
18:1(n-7)	3.40±0.51	2.62±0.20	1.43±0.09	1.69±0.24	6.80±0.65
20:1(n-9) tr*	0.02±0.01	0.04±0.01	0.03±0.01	0.03±0.02	0.02±0.02
20:1(n-9)	2.14±0.29	1.92±0.40	1.91±0.34	2.44±0.30	1.72±0.11
22:1(n-9)	0.08±0.04	0.13±0.07	0.10±0.06	0.15±0.07	0.08±0.03
24:1(n-9)	0.00±0.00	0.02±0.01	0.01±0.01	0.01±0.01	0.00±0.00
Polyunsaturated					
n-3					
18:3(n-3)	0.18±0.08	0.20±0.07	0.08±0.04	0.13±0.05	0.29±0.12
18:4(n-3)	0.51±0.08	0.39±0.06	0.27±0.03	0.54±0.04	0.51±0.06
20:5(n-3)	0.24±0.09	0.08±0.04	0.03±0.01	0.15±0.04	0.15±0.03
22:5(n-3)	0.01±0.01	0.04±0.01	0.09±0.01	0.00±0.00	0.00±0.00
22:6(n-3)	0.08±0.02	0.23±0.07	0.55±0.07	0.13±0.03	0.06±0.01
n-6					
18:2 (n-6)	2.33±0.09	6.60±0.47	3.73±0.18	22.50±0.71	1.56±0.16
18:3(n-6)	0.05±0.02	0.21±0.03	0.66±0.07	0.11±0.01	0.04±0.02
20:2(n-6)	0.06±0.01	0.11±0.03	0.40±0.07	0.06±0.01	0.45±0.04
20:3(n-6)	0.14±0.04	0.40±0.13	1.08±0.18	0.21±0.04	0.00±0.00
20:4(n-6)	0.20±0.04	0.39±0.07	1.00±0.19	0.27±0.06	0.15±0.01
20:4(n-6)	0.20±0.04	0.39±0.07	1.00±0.19	0.27±0.06	0.15±0.01
22:4(n-6)	0.07±0.01	0.12±0.03	0.30±0.08	0.06±0.02	0.05±0.01
22:5(n-6)	0.08±0.03	0.11±0.05	0.33±0.15	0.08±0.03	0.01±0.01
n-9					
18:2(n-9)	0.15±0.01	0.21±0.01	0.21±0.00	0.23±0.01	0.12±0.01
20:3(n-9)	0.67±0.08	0.30±0.02	0.13±0.01	0.51±0.07	0.09±0.02
ΣSFA	23.1±1.0	18.0±0.8	14.3±0.2	15.1±0.4	25.3±0.8
ΣMUFA	70.6±0.8	70.9±0.6	77.3±0.6	55.6±1.0	69.2±0.8
ΣPUFA	3.6±0.2	8.5±0.7	5.0±0.3	25.0±1.0	2.5±0.3
ΣHUFA	1.2±0.3	1.3±0.3	1.2±0.2	2.5±0.5	0.9±0.1
16:1n7/16:0-ratio	0.11±0.01	0.12±0.01	0.10±0.01	0.08±0.01	0.18±0.01
18:1n9/18:0-ratio	24.5±2.3	47.9±2.2	62.3±3.0	33.3±3.0	31.3±2.5
n3/n6-ratio	1.9±0.16	0.7±0.07	1.4±0.11	0.4±0.05	1.5±0.21

* tr=trans fatty acid
